# Egg envelope formation of medaka *Oryzias latipes* requires ZP proteins originating from both the liver and ovary

**DOI:** 10.1016/j.jbc.2023.104600

**Published:** 2023-03-10

**Authors:** Reo Yokokawa, Kana Watanabe, Shinji Kanda, Yoshihide Nishino, Shigeki Yasumasu, Kaori Sano

**Affiliations:** 1 Department of Materials Science, Graduate School of Science, Josai University, Sakado, Japan; 2 Graduate School of Pharmaceutical Sciences, Josai University, Sakado, Japan; 3 Atmosphere and Ocean Research Institute, The University of Tokyo, Kashiwa, Japan; 4 Department of Materials and Lifesciences, Faculty of Science and Technology, Sophia University, Tokyo, Japan; 5 Department of Chemistry, Faculty of Science, Josai University, Sakado, Japan

**Keywords:** gene knockout, gene expression, protein synthesis, protein domain, protein cross-linking, ZP protein, choriogenin, chorion, egg envelope

## Abstract

Teleost oocytes are surrounded by a structure called chorion or egg envelopes, which is composed of zona pellucida (ZP) proteins. As a result of the gene duplication in teleost, the expression site of the *zp* genes, coding the major component protein of egg envelopes, changed from the ovary to the maternal liver. In Euteleostei, there are three liver-expressed *zp* genes, named *choriogenin* (*chg*) *h*, *chg hm*, and *chg l*, and the composition of the egg envelope is mostly made up of these Chgs. In addition, ovary-expressed *zp* genes are also conserved in the medaka genomes, and their proteins have also been found to be minor components of the egg envelopes. However, the specific role of liver-expressed *versus* ovary-expressed *zp* genes was unclear. In the present study, we showed that ovary-synthesized ZP proteins first form the base layer of the egg envelope and then Chgs polymerize inwardly to thicken the egg envelope. To analyze the effects of dysfunction of the *chg* gene, we generated some *chg* knockout medaka. All knockout females failed to produce normally fertilized eggs by the natural spawning. The egg envelopes lacking Chgs were significantly thinner, but layers formed by ZP proteins synthesized in the ovary were found in the thin egg envelope of knockout as well as wildtype eggs. These results suggest that the ovary-expressed *zp* gene is well conserved in all teleosts, including those species in which liver-derived ZP proteins are the major component, because it is essential for the initiation of egg envelope formation.

Vertebrate oocytes are surrounded with a proteinaceous structure called “zona pellucida” in mammals, “vitelline membrane” in birds, “vitelline envelope” in amphibians, and “egg envelope” or “chorion” in fish (1, 2, 3, 4, 5, 6). These structures are commonly formed by polymerization of the zona pellucida (ZP) domains, which are 260 to 280 amino acids in length, that the ZP protein possesses. ZP proteins are classified into ZPA, ZPB, ZPC, ZPD, and ZPAX based on amino acid sequence homology in the ZP domain (7). Mammalian ZP consists of ZPA, B, and C, while the amphibian vitellin envelope is composed of ZPA, ZPB, ZPC, ZPD, and ZPAX. The egg envelope of teleosts is composed of ZPB, ZPC, and ZPAX; the ZPA gene has been lost from their genomes (8). Thus, despite differences in the subtypes of ZP proteins that contribute to composition, the ZP proteins are well conserved in vertebrates and some invertebrates (9). In many cases, *zp* genes are expressed in growing oocytes in the ovary, and some are expressed in the liver.

In teleosts, the expression site of the *zp* genes that constitute the main component of the egg envelope has changed from the ovary to the maternal liver during evolution. In Elopomorpha and Osteogrossomorpha, the most ancient group of teleosts, *zp* genes are expressed in the ovary (oocyte) (10). In the common ancestor of Euteleostei and Otocephala, the *zp* genes were duplicated, and liver-expressed ZP genes appeared (11, 12, 13, 14). Although the detailed transport mechanism is still unknown, ZP proteins synthesized in the maternal liver were carried to the ovary through the bloodstream and formed the egg envelope around growing oocytes (15, 16, 17, 18, 19, 20). The egg envelopes of Euteleostei and Clupeiformes belonging to Otocephala were thicker and tougher. Interestingly, the timing of the acquisition of *zp* genes expressed in liver, which can synthesize large amounts of the protein, coincides with the timing of acquisition of thick egg envelopes (13, 21). In Euteleostei, liver-expressed *zp* genes are maintained and named choriogenin (Chg); there are three types of Chgs: ChgH and ChgHm, which are classified into ZPB, and ChgL, which is classified into ZPC (14, 22, 23). In Otocephala, the liver-expressed *zp* genes disappeared after the divergence of Clupeiformes (13, 21). Therefore, otophysian fishes, which are included in Otocephala, synthesize egg envelopes in their growing oocytes (24, 25, 26, 27).

Interestingly, in addition to Chgs, the other *zp* genes expressed in the ovary, *zpax*, *zpb*, and *zpc*, were conserved in all Euteleostei genomes, although their volume in the egg envelope is much smaller than the liver-expressed *chg*s. Therefore, there are three *chg* genes (*chg h*, *chg hm*, *chg l*) and eight ovary-expressed *zp* genes (*zpax1*, *zpax2*, *zpb*, *zpc1-5*) in the medaka, *Oryzias latipes*, genome (28, 29). It is also suggested that the *chg* genes, especially *chg h* and *chg hm*, have evolved independently from those of tetrapods (14). ChgH and Hm proteins have a long three-amino-acid repeat sequence (Pro-Gln-Xaa repeat) at the N-terminal side of the ZP domain (21, 23). At fertilization, hardening is carried out when the transglutaminase localized in the egg envelope catalyzes the formation of ε-(γ-glutamyl) lysine isopeptide cross-links between ZP proteins (30). Most of the cross-links are formed between the N-terminal Pro-Gln-Xaa repeat regions of ChgH and Hm. At hatching, one of the hatching enzymes, high choriolytic enzyme, swells and softens the egg envelope by cleaving the Pro-Gln-Xaa repeat regions into small peptides that are released from the egg envelope, and then another enzyme, low choriolytic enzyme, solubilizes it completely (23, 31). Therefore, N-terminal regions of ChgH and Hm, the structure of which are not found in other vertebrate ZP proteins, are the substrates of transglutaminase and the hatching enzyme and result in teleost-specific molecular evolution.

Recently, it was reported that a null mutant of the medaka *chg l* gene has been generated in which the eggs have a thin egg envelope and are infertile when artificially fertilized with wildtype (WT) sperm and that the ovary-expressed *zp* gene cannot compensate for the deficiency in liver-expressed *chg l* (32, 33). However, although the timing of the presence of each ZP protein was indicated in that report, detailed localization was not provided. In this study, we generated ChgH knockout (ko) (*chg h*^*−/−*^) and two kinds of double ko (*chg h*^*−/−*^*; chg hm*^*−/−*^ and *chg h*^*−/−*^*; chg l*^*−/−*^) medaka to analyze the effect of dysfunction of the *chg h* gene, the most characteristic ZP protein in Euteleostei, on egg envelope formation. Moreover, we examined the localization of ovary-synthesized ZP proteins in WT and the three ko medaka, and showed that the localization of ovary-expressed ZP proteins changes as the oocytes grow. Finally, we infer the role of *chg* genes and ovary-expressed *zp* genes in egg envelope formation and discuss evolutionary considerations related to the egg envelope formation of ZP proteins.

## Results

### Egg envelope formation process of medaka

**Figure 1:**
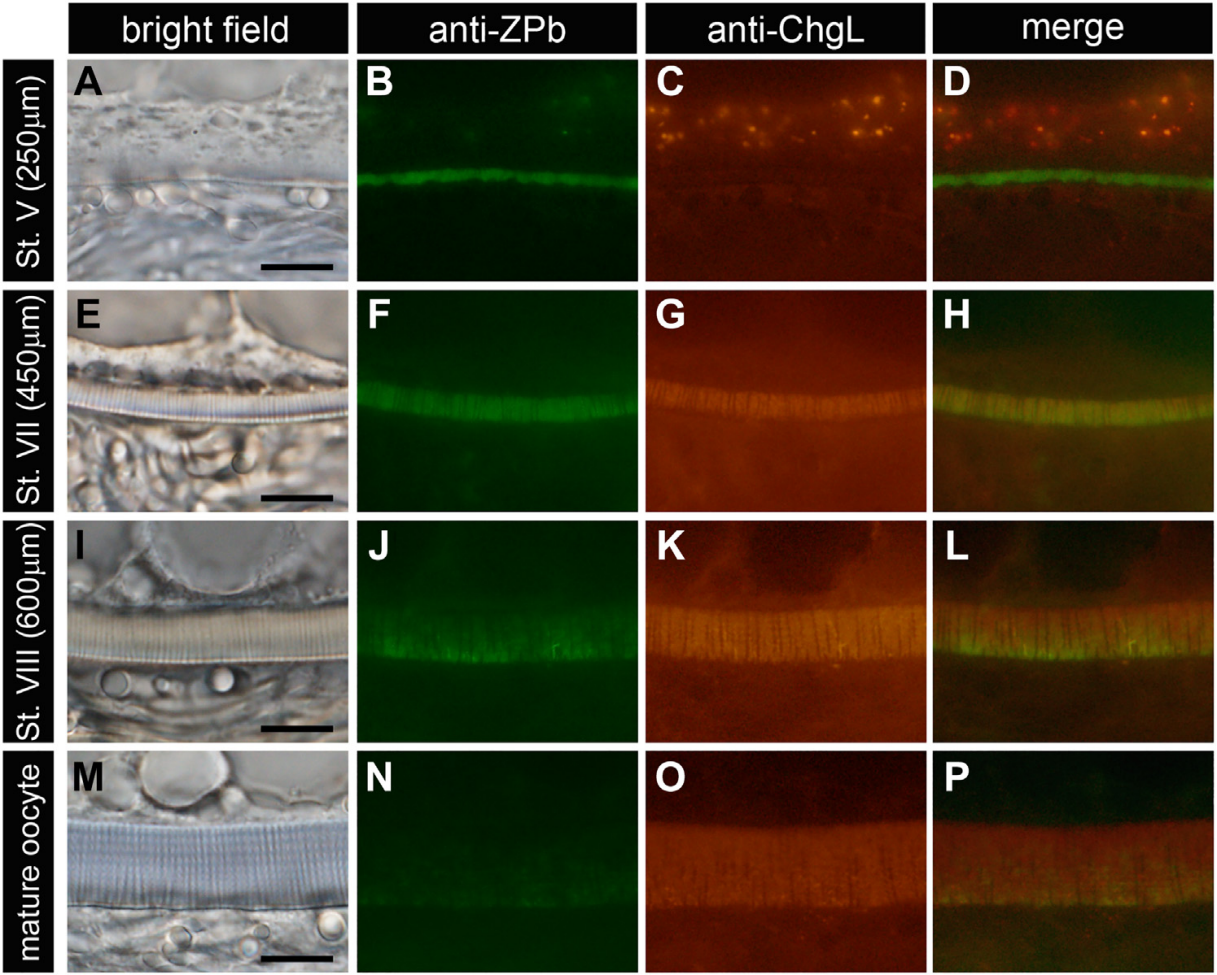
**Localization of ChgL and ZPb in the egg envelope of WT growing oocytes.** Localization of ChgL and ZPb was analyzed by double-labeled immunohistochemistry using anti-ChgL and anti-ZPb antibodies. *A*–*D*, St. V oocyte (250 μm). *E*–*H*, St. VII oocyte (450 μm). *I*–*L*, St. VIII oocyte (600 μm). *M*–*P*, mature oocyte. The *upper side* of all photos is the cytoplasmic side. The scale bars represent 10 μm.

**Figure 2:**
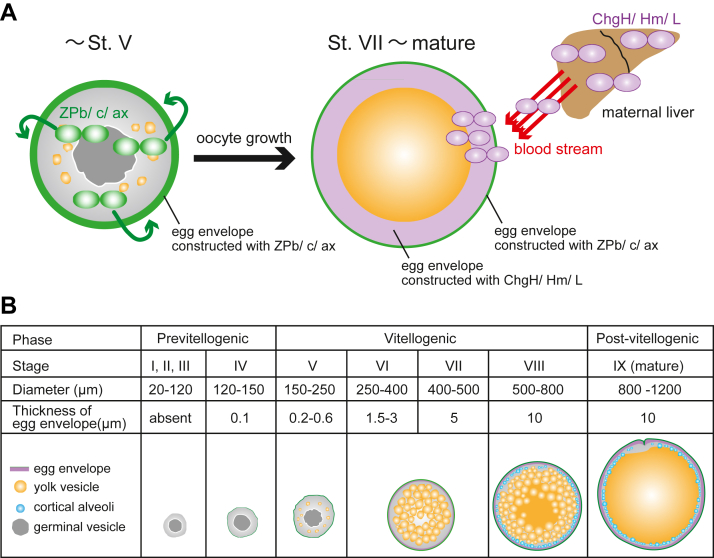
**Schematic diagram of the egg envelope formation process.***A*, the predicted process of egg envelope formation at oogenesis is shown. The *yellow circles* indicate the oocyte. The *green* and *purple ovals* indicate the oocyte-synthesized and liver-synthesized ZP protein, respectively. The *green layer* is the egg envelope formed by oocyte-synthesized ZP protein (like ZPb, ZPc1-5, ZPAX1, 2). The *purple layer* is the egg envelope formed by liver-derived ZP protein (ChgH, Hm, L). *B*, the oogenesis process of medaka is shown with a specific focus on the egg envelope formation (34). Although follicle cells and attaching filaments are essentially present further out in the egg envelope, they are excluded in this illustration.

First, the procedure of the accumulation of ZP proteins in the egg envelope during oogenesis of WT medaka was observed by the immunohistochemical method. The section of ovary containing various-stage oocytes was labeled by anti-ZP protein antibodies. We employed anti-ChgL, ChgH, and ChgHm antibodies for the detection of liver-derived ZP proteins and anti-ZPb, ZPc5, and ZPAX1 antibodies for the detection of ovary-synthesized ZP proteins. To clearly indicate the subtle differences in localization, double-label immunohistochemistry was performed on the same sections for ovary- and liver-expressed *zp* genes. In stage (St.) V oocytes (250 μm in diameter), ZPb protein was localized in a thin egg envelope in the early choriogenesis stage, whereas no ChgL was detected (Fig. 1, *A*–*D*). In St. VII oocytes, both ZPb and ChgL were located throughout the entire egg envelope (Fig. 1, *E*–*H*). In St. VIII to mature oocytes, ChgL was located throughout the entire egg envelope; however, ZPb was found only in the outermost layer of the egg envelope (Fig. 1, *I*–*P*). The localization patterns of other liver-derived ZP proteins (ChgH and Hm) were similar to that of ChgL (Fig. S1, *A* and *B*). In the other ovary-synthesized ZP proteins, ZPc5 showed a similar localization pattern to ZPb (Fig. S1*A*). The case of ZPAX1, however, was somewhat different: ZPAX1 was localized to the egg envelope from the early choriogenesis stage, and then, even after Chgs occupied most of the egg envelope in stage VIII and mature oocytes, ZPAX1 also localized especially on the inner side of the egg envelope (Fig. S1*B*). This result suggests that the role of ZPAX in egg envelope formation was different from that of other ovary-synthesized ZP proteins, ZPb and ZPc. It was reported that anti-ZPc5 antibody was detected in the egg envelope in the early stage of choriogenesis but not in the mature egg envelope (33). However, our results in the present study indicate that ZPb and ZPc5 proteins are slightly localized in the outermost layer of the mature egg envelope. From the above results, the egg envelope formation process can be inferred as follows. The ovary-synthesized ZP proteins predominantly accumulate in the egg envelope in the early stage of choriogenesis, and then liver-derived ZP proteins, Chgs, accumulate on the inside of the layer composed of ovary-synthesized ZP proteins to form a thick egg envelope. As a result, the ovary-synthesized ZP proteins, like ZPb and ZPc5, are pushed out to the periphery. We summarized the hypothetical mechanism of the formation of egg envelope from ovary- and liver-derived ZP proteins in Figure 2 (34). Based on this hypothesis, to understand the role of liver-derived ZP proteins in the egg envelope formation, we generated ChgH ko (*chg h*^*−/−*^) and two double ko (*chg h*^*−/−*^*; chg hm*^*−/−*^ and *chg h*^*−/−*^*; chg l*^*−/−*^) medaka.

### Establishment of ChgH ko (*chg h*^−/−^) and double ko (*chg h*^*−/−*^*; chg hm*^*−/−*^ and *chg h*^*−/−*^*; chg l*^*−/−*^) strains

CRISPR guide RNAs were designed immediately after the initiation codons of *chg h, hm* and *l*, and the guide RNAs were injected with Cas9 nuclease in the one- or two-cell stage of d-rR medaka eggs. After breeding with WT d-rR strain, the obtained heterozygous mutants were further bred with each other. Sequence analysis was performed on the target genes, and null mutants were obtained. Cas9 cleavage of the target site of each gene resulted in inducing a frameshift and a stop codon early in the reading frame of *chg h*, *chg hm*, and *chg l* genes (Fig. S2). In the *chg h*^−/−^; *chg hm*^−/−^ double ko medaka, 3864 bp of large deletion spanning from −1988 bp to the fourth exon of *chg h* was detected as a result of the injection of a mixture of *chg h*, *chg hm* guide RNAs (Fig. S2). Since ChgH ko and both double ko (*chg h*^*−/−*^; *chg hm*^*−/−*^, and *chg h*^*−/−*^*; chg l*^*−/−*^) females were infertile, these strains were established by breeding null mutant males and heterozygous mutant females to obtain offspring.

### Expression analysis of Chg and other ZP genes by real-time PCR

**Figure 3:**
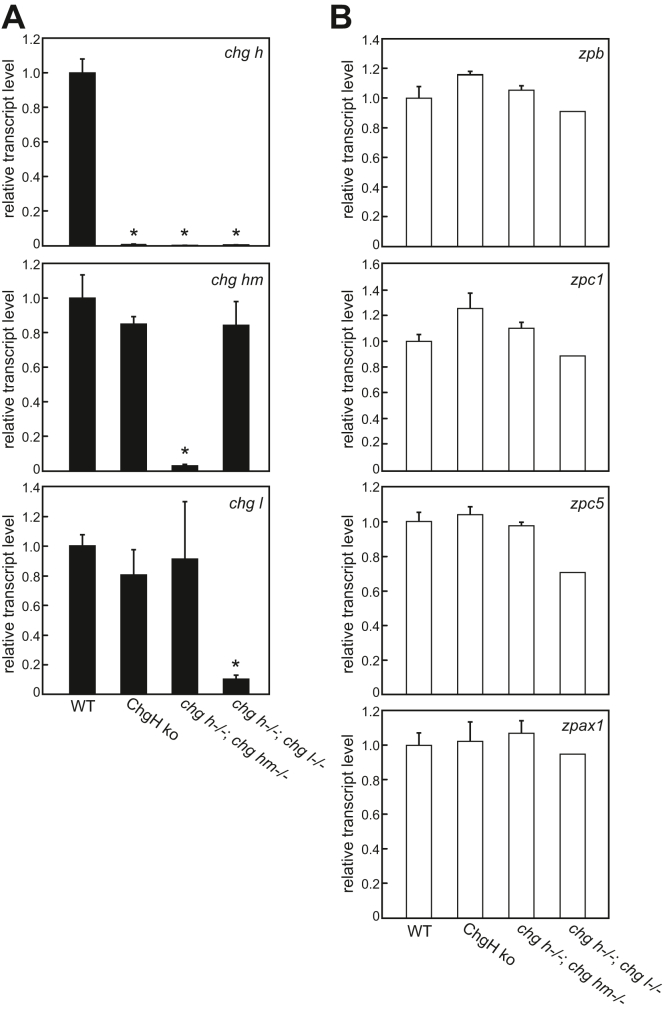
**Quantitative PCR analyses of liver-expressed *chg* genes and ovary-expressed *zp* genes.** Transcript levels of each gene of WT, ChgH ko, *chg h*^−/−^; *chg hm*^−/−^ and *chg h*^−/−^; *chg l*^−/−^ double ko were compared. Expression was normalized against that of *β-actin* gene. The relative transcript level of each gene was calculated according to the comparative Ct method, with the expression level of each gene in WT set to 1. *A*, the transcript levels of *chg h*, *chg hm*, and *chg l* genes in the maternal liver. Each experiment was performed using RNA extracted from three different individuals (n = 3). *Asterisks* indicate a significant difference (*p* < 0.05) compared with WT as determined by Welch’s *t* test. *B*, the transcript levels of the *zpb*, *zpc1*, *zpc5*, and *zpax1* genes in the ovary contained only St. I-VII oocytes, excluding mature oocytes (WT, ChgH ko, *chg h*^−/−^; *chg hm*^−/−^: n = 3, *chg h*^−/−^; *chg l*^−/−^: n = 2)

The expression levels of the *chg* genes in the liver of mature females of ChgH ko, double ko, and WT were compared (Fig. 3*A*). The expression level of the *chg h* gene in the liver of ChgH ko females was significantly reduced compared with that of WT, while the expression level of *chg hm* and *chg l* genes did not differ from WT. In *chg h*^*−/−*^*; chg hm*^*−/−*^ double ko females, the expression level of *chg h* and *chg hm* genes was significantly decreased and that of the *chg l* gene did not differ from that of the WT. In *chg h*^*−/−*^*; chg l*^*−/−*^ double ko females, the expression level of *chg h* and *chg l* genes was significantly decreased and that of the *chg hm* gene did not differ from that of the WT. In addition, the expression level of *zpb*, *zpc1*, *zpc5*, and *zpax1* genes in immature oocytes was compared among WT and ko (Fig. 3*B*). As a result, there was no significant difference in the expression levels of those genes between them. These results suggest that no compensation of either liver-expressed or ovary-expressed *zp* genes occurred in ChgH ko and double ko individuals.

### Analysis of egg envelope components by Western blotting

**Figure 4:**
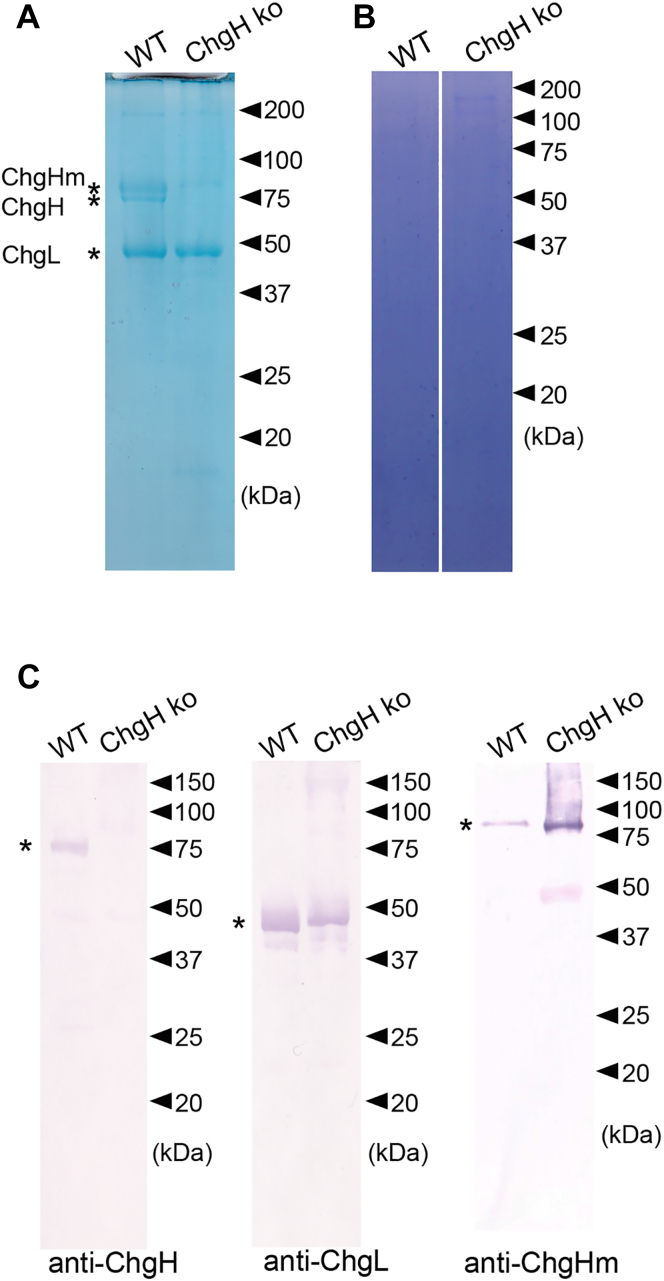
**SDS-PAGE and Western blotting of WT and ChgH ko egg envelopes.** Unfertilized egg envelope (*A*) and naturally spawned egg envelope (*B*) were loaded onto a 12.5% polyacrylamide gel. The gel was stained by Coomassie Brilliant Blue-250. *C*, unfertilized egg envelopes were loaded onto a 12.5% polyacrylamide gel, and each of the Chgs were detected using antibodies. *Asterisks* indicate the band position of each Chg in the WT.

Egg envelopes isolated from ovulated eggs from ChgH ko and WT were called “unfertilized egg envelopes” (UFEs) and used for SDS-PAGE. As indicated by asterisks, the SDS-PAGE pattern of WT UFEs showed individual bands of ChgHm (77 kDa), H (75 kDa), and L (48 kDa). However, that of ChgH ko UFEs showed ChgHm and L bands but not the chgH band (Fig. 4*A*). These results were consistent with that of *chg* gene expression in mature female livers. Next, eggs from the abdomen of naturally spawned females of ChgH ko and WT were obtained, and the egg envelopes isolated from them were used for SDS-PAGE as “naturally spawned egg envelopes” (NSEs). Since egg envelopes become hardened after fertilization by the formation of ε-(γ-glutamyl) lysine isopeptide cross-links and are insoluble in SDS, no bands were obtained in the NSE of the WT (Fig. 4*B*). Similarly, no bands were found in the NSE of ChgH ko (Fig. 4*B*). These results suggest that, even though ChgH ko is unable to produce normal fertilized eggs by natural spawning, the hardening process of the egg envelopes occurred similarly to WT.

To confirm the constituent proteins of the egg envelopes, Western blot analysis of UFEs was performed using anti-ChgH, Hm, and L antibodies (Fig. 4*C*). As indicated by asterisks, the three antibodies each detected specific bands in WT UFEs. ChgH ko UFE had bands that reacted with anti-ChgHm and ChgL antibodies but not with the anti-ChgH antibody (Fig. 4*C*). UFEs of *chg h*^*−/−*^*; chg hm*^*−/−*^ and *chg h*^*−/−*^*; chg l*^*−/−*^ double ko were too fragile to isolate alone.

### Natural spawning observation of ko strains

**Figure 5:**
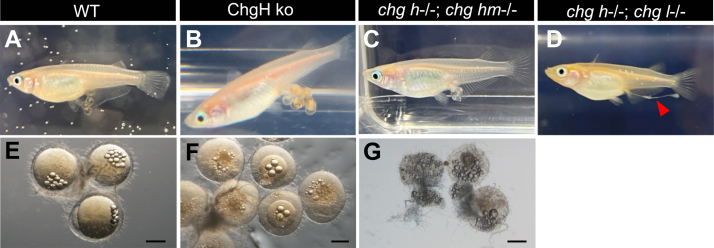
**Observation of natural spawning and eggs obtained from the abdomen of females.** Mature females of WT (*A*), ChgH ko (*B*), *chg h*^−/−^; *chg hm*^−/−^ (*C*), and *chg h*^−/−^; *chg l*^*−/−*^ (*D*) were mated with WT males and observed to spawn naturally. Eggs isolated from the abdomen of females of WT (*E*), ChgH ko (*F*), and *chg h*^−/−^; *chg hm*^−/−^ (*G*). The scale bars in (*E*–*G*) represent 500 μm.

To observe natural spawning, each of the ko and WT mature females were mated with WT d-rR males. On the next morning, eggs were found on the abdomen of ChgH ko, *chg h*^−/−^; *chg hm*^−/−^ double ko, and WT females (Fig. 5, *A*–*C*), but only collapsed eggs were found on the abdomen of *chg h*^−/−^; *chg l*^−/−^ double ko females, and no spherical eggs were observed (Fig. 5*D*). The eggs were isolated from the abdomen of ChgH ko, *chg h*^−/−^; *chg hm*^−/−^ double ko and WT female (Fig. 5, *E*–*G*). Although both ko eggs had an egg envelope, they were soft and easily tearing with tweezers, especially in the double ko. Furthermore, they were not as spherical as the WT eggs and the double ko eggs in particular appeared friable and had lost some of their yolks (Fig. 5, *E*–*G*). Subsequent observations revealed that neither of the ko eggs had cell division or embryogenesis. These results suggest that ChgH ko and both double ko (*chg h*^*−/−*^*; chg hm*^*−/−*^ and *chg h*^*−/−*^*; chg l*^*−/−*^) females were unable to produce normally fertilized eggs in natural spawning, since the eggs would be broken by mechanical stress while passing through the oviduct. Considering that the eggs were collapsed during spawning, artificial insemination of the ovulated eggs was attempted.

### Artificial fertilization of ChgH ko and double ko eggs

**Figure 6:**
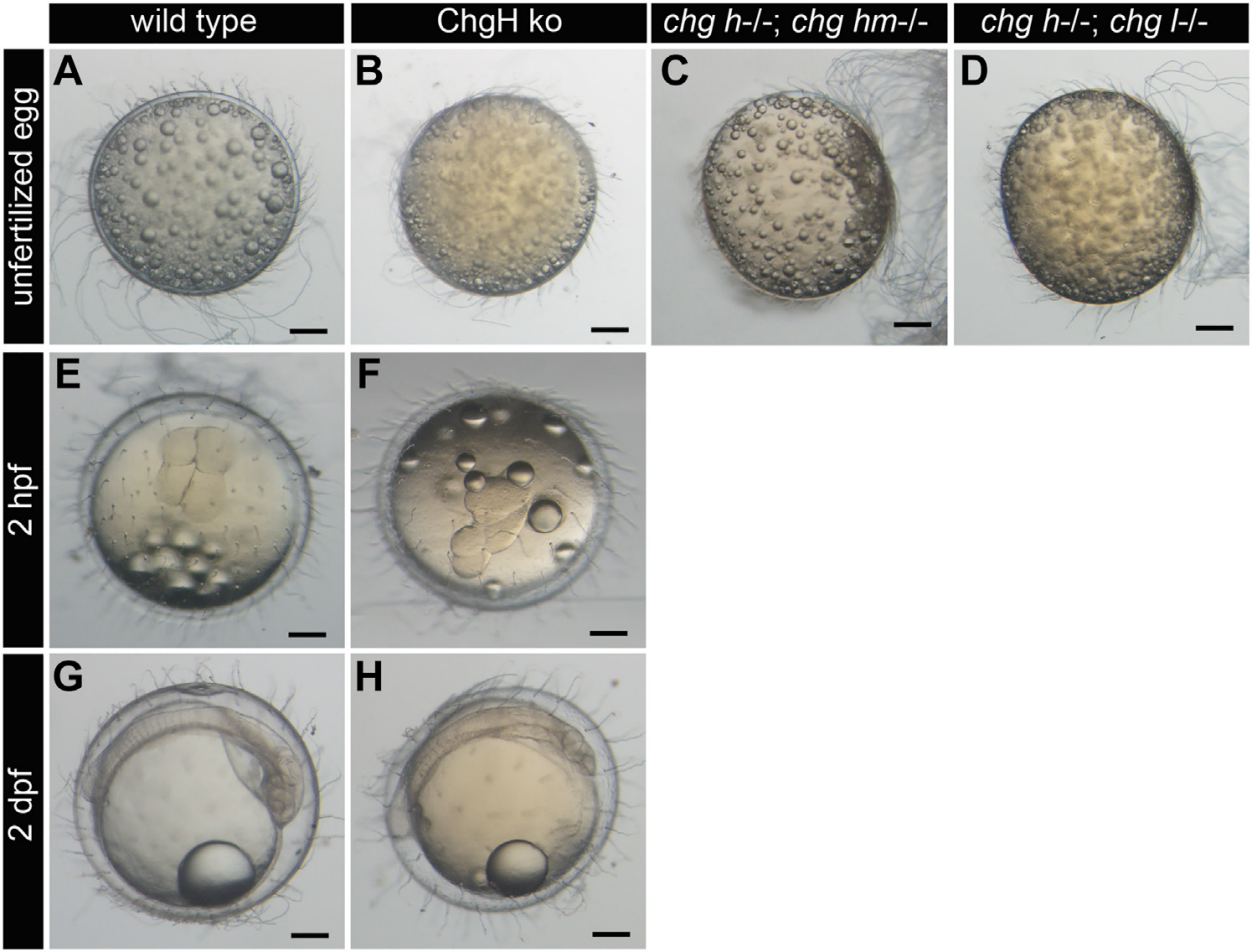
**WT and ko unfertilized eggs and their artificial insemination.** Ovulated eggs of WT (*A*), ChgH ko (*B*), *chg h*^−/−^; *chg hm*^−/−^ (*C*), and *chg h*^−/−^; *chg l*^−/−^ (*D*) collected from the female abdominal cavity. Observation of fertilized eggs of WT (*E* and *G*) and ChgH ko (*F* and *H*) 2 h and 2 days after artificial insemination. The scale bars represent 200 μm.

The ovulated eggs isolated from the body cavity of WT, ChgH ko, and both double ko (*chg h*^*−/−*^*; chg hm*^*−/−*^ and *chg h*^*−/−*^*; chg l*^*−/−*^) females were *in vitro* fertilized with sperm from WT males. Observation of the ovulated eggs before insemination revealed that the eggs of the ChgH ko were similar in appearance to those of the WT, but the eggs of both double ko failed to maintain a spherical shape and appeared slightly oval spheroid (Fig. 6, *A*–*D*). In WT and ChgH ko eggs, the cortical alveoli breakdown and the formation of the perivitelline space was observed immediately after the addition of the sperm suspension (18/20 eggs). However, many ChgH ko embryos had no or abnormal egg cleavage (17/18 embryos, Table S1). This phenomenon may be attributed to polyspermy. Only one ChgH ko embryo was observed to have 4-cell stage and embryo axis formation (Fig. 6, *F* and *H*). These results indicate that ChgH ko eggs are fertilizable but do not have a complete polyspermy block mechanism. In both double ko eggs, no perivitelline space was formed and no oil droplet migration was observed after the addition of sperm suspension; that is, fertilization was not possible.

### Ovarian morphology of ChgH ko and double ko

**Figure 7:**
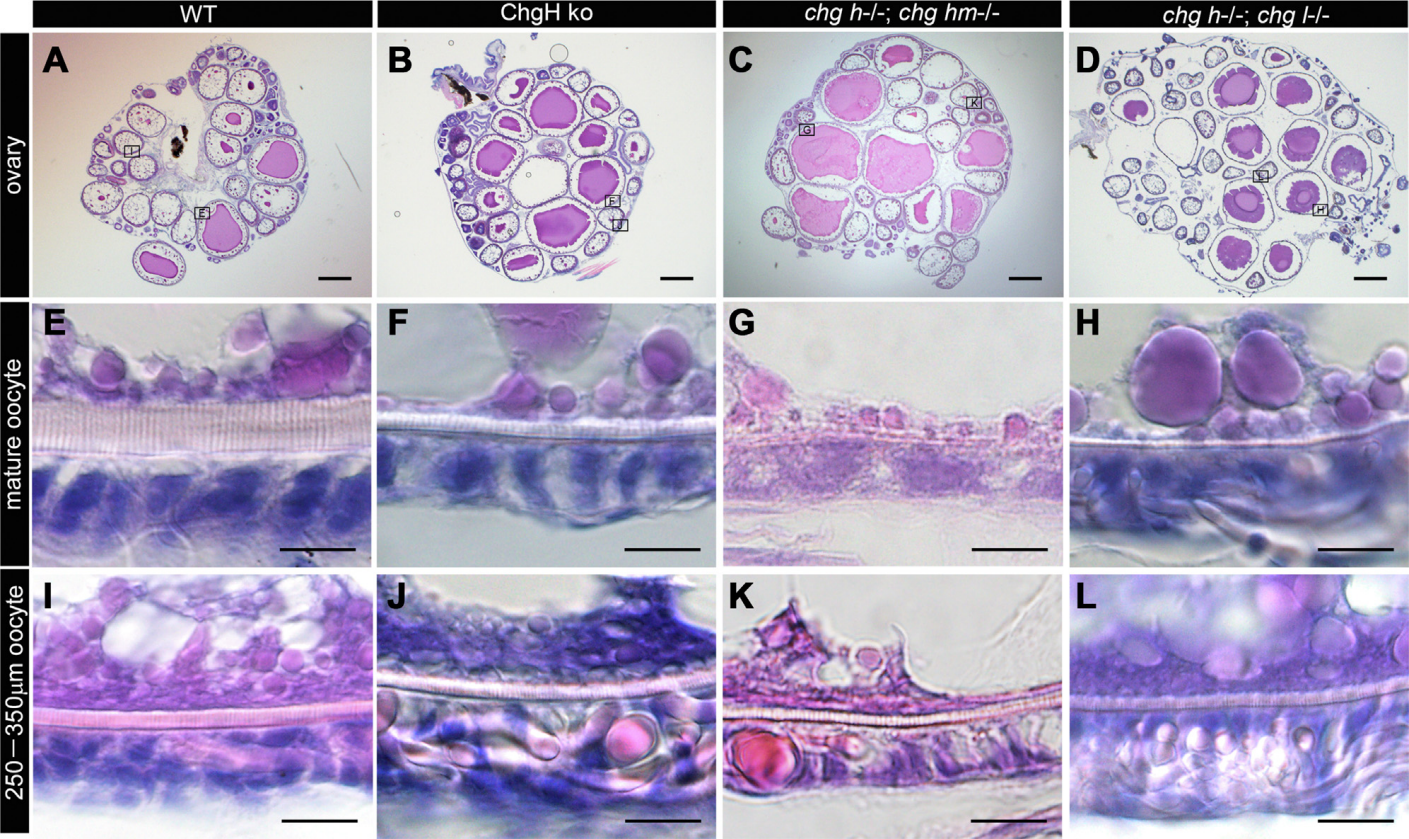
**H&E staining of ovaries.** The section of whole ovaries of WT (*A*), ChgH ko (*B*), *chg h*^−/−^; *chg hm*^−/−^ (*C*), and *chg h*^−/−^; *chg l*^−/−^ (*D*). Zoomed-in views of the oocytes in *squares* with the alphabet in the photos of the whole ovaries are in the photos of mature (*E*–*H*) and immature oocytes (*I*–*L*). The *upper* side of the zoomed-in view photos is the cytoplasmic side. The scale bars represent in (*A*–*D*): 500 μm, and (*E*–*L*): 10 μm.

To compare the ovarian morphology, ovarian sections stained by hematoxylin and eosin (H&E) were observed. All ovaries contained various stages of oocytes, suggesting that the oogenesis of ChgH ko and double ko was normal (Fig. 7, *A*–*D*). Comparing mature oocytes, the egg envelope of the ChgH ko oocyte was thinner than that of the WT and that of both double ko oocytes was even thinner (Fig. 7, *E*–*H*). The thickness of the egg envelopes of St. V-VI, VIII and mature oocytes was estimated using ImageJ software (Table 1). The thickness of the egg envelope of mature oocytes was determined to be 2.84 ± 0.47 μm for ChgH ko, 0.70 ± 0.07 μm for *chg h*^*−/−*^*; chg hm*^*−/−*^ double ko, and 1.50 ± 0.07 μm for *chg h*^*−/−*^*; chg l*^*−/−*^ double ko, which were much thinner than WT (12.9 ± 3.83 μm). Interestingly, no significant difference was observed between them when comparing the thickness of the egg envelopes of immature oocytes of St. V-VI (Fig. 7, *I*–*L* and Table 1).

**Table 1:** Thickness of egg envelopes estimated from the ovary sections by ImageJ

Genotype	St. V-VI	St. VIII	Mature
WT	2.84 ± 0.55	4.12 ± 0.14	12.9 ± 3.83
*chg h*^*−/−*^	2.41 ± 0.22	3.87 ± 0.90	2.84 ± 0.47
*chg h*^*−/−*^*; chg hm*^*−/−*^	2.16 ± 0.10	0.92 ± 0.03	0.70 ± 0.07
*chg h*^*−/−*^*; chg l*^*−/−*^	2.56 ± 0.19	2.04 ± 0.34	1.50 ± 0.07

The thickness of the egg envelope in stage (St.) V-VI (250–350 μm in diameter), St. VIII (600–800 μm in diameter), and mature oocyte (1000–1200 μm in diameter) was measured at four locations (n = 4).

### Determination of total protein content of egg envelopes

**Figure 8:**
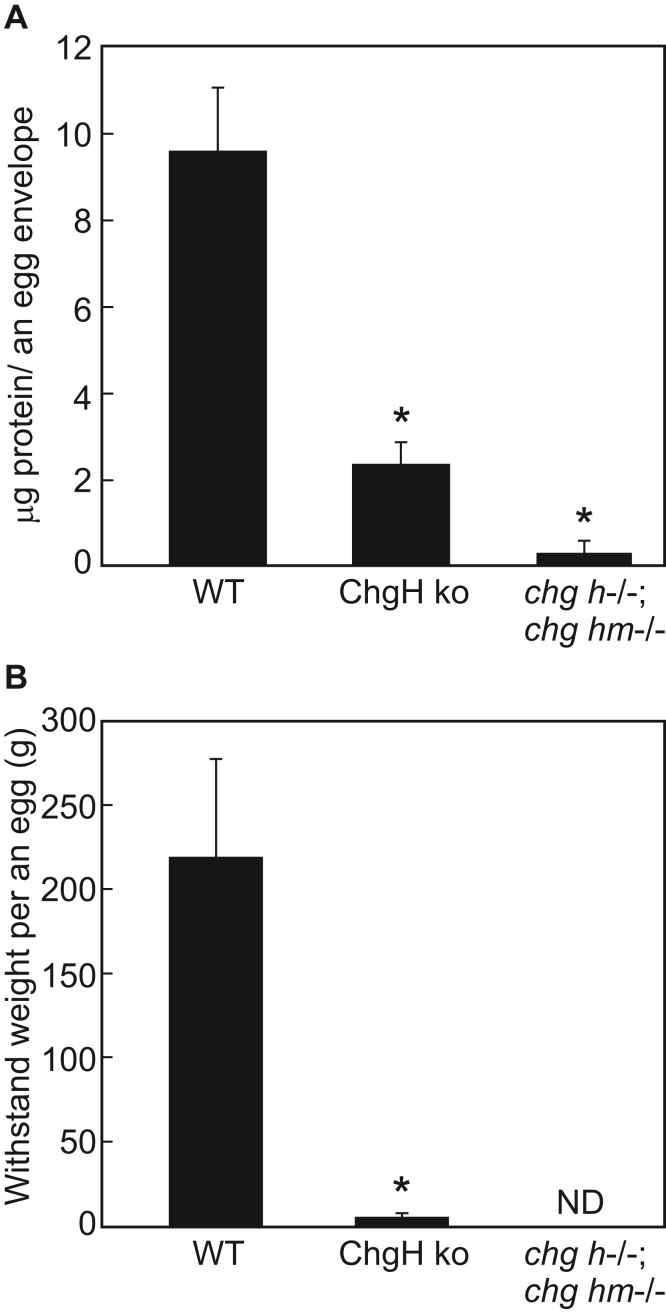
**Total protein content and toughness of egg envelope.***A*, total protein content per egg envelope, estimated by the Bradford method after complete solubilization of the isolated egg envelope with hatching enzymes. *Asterisks* indicate significant difference (*p* < 0.05) compared with WT as determined by Welch’s *t* test (WT: n = 5, ChgH ko: n = 6, *chg h*^−/−^; *chg hm*^−/−^: n = 3). *B*, toughness of eggs were compared. (WT: n = 7, ChgH ko: n = 5, *chg h*^−/−^; *chg hm*^−/−^ n = 3)

The amounts of total protein comprising the egg envelopes of ChgH ko *chg h*^*−/−*^*; chg hm*^*−/−*^ double ko, and WT were compared. The egg envelopes were isolated from eggs on the abdomen of a natural spawning female and solubilized by medaka hatching enzymes. The inner layer composed of ZP proteins was solubilized, and the thin outer layer that was not digested by the hatching enzymes was removed by centrifugation (23, 35). Then, the total protein content of the supernatant was determined by a Bradford assay. As a result, the total protein content of the egg envelope per egg for WT, ChgH ko, and *chg h*^*−/−*^*; chg hm*^*−/−*^ double ko was approximately 9.5, 2.3, and 0.3 μg, respectively (Fig. 8*A*). This indicated that the loss of a single *chg h* gene results in only 24% the amount of the egg envelope protein per egg compared with the WT. Furthermore, when both *chg h* and *chg hm* genes were lost, the eggs accumulated only 3.2% the amount of ZP protein as compared with WT eggs.

### Comparison of the toughness of eggs

To quantify the toughness of eggs, we measured the minimum weight that can squash a whole egg. Eggs spawned by WT, ChgH ko, and *chg h*^*−/−*^*; chg hm*^*−/−*^ double ko females were collected from the abdomen and weighed to compare how much weight they could bear. WT eggs could withstand a weight of 219.7 ± 58.0 g, but ChgH ko eggs ruptured easily after only 5.7 ± 2.2 g (Fig. 8*B* and Table S2). Thus, it was suggested that thin egg envelopes lacking ChgH were approximately 1/40th the strength of the WT. *chg h*^*−/−*^*; chg hm*^*−/−*^ double ko eggs were too brittle to measure (Fig. 8*B*).

### Localization analysis of liver-derived Chg and ovary-synthesized ZP proteins by double-labeled immunohistochemistry

**Figure 9:**
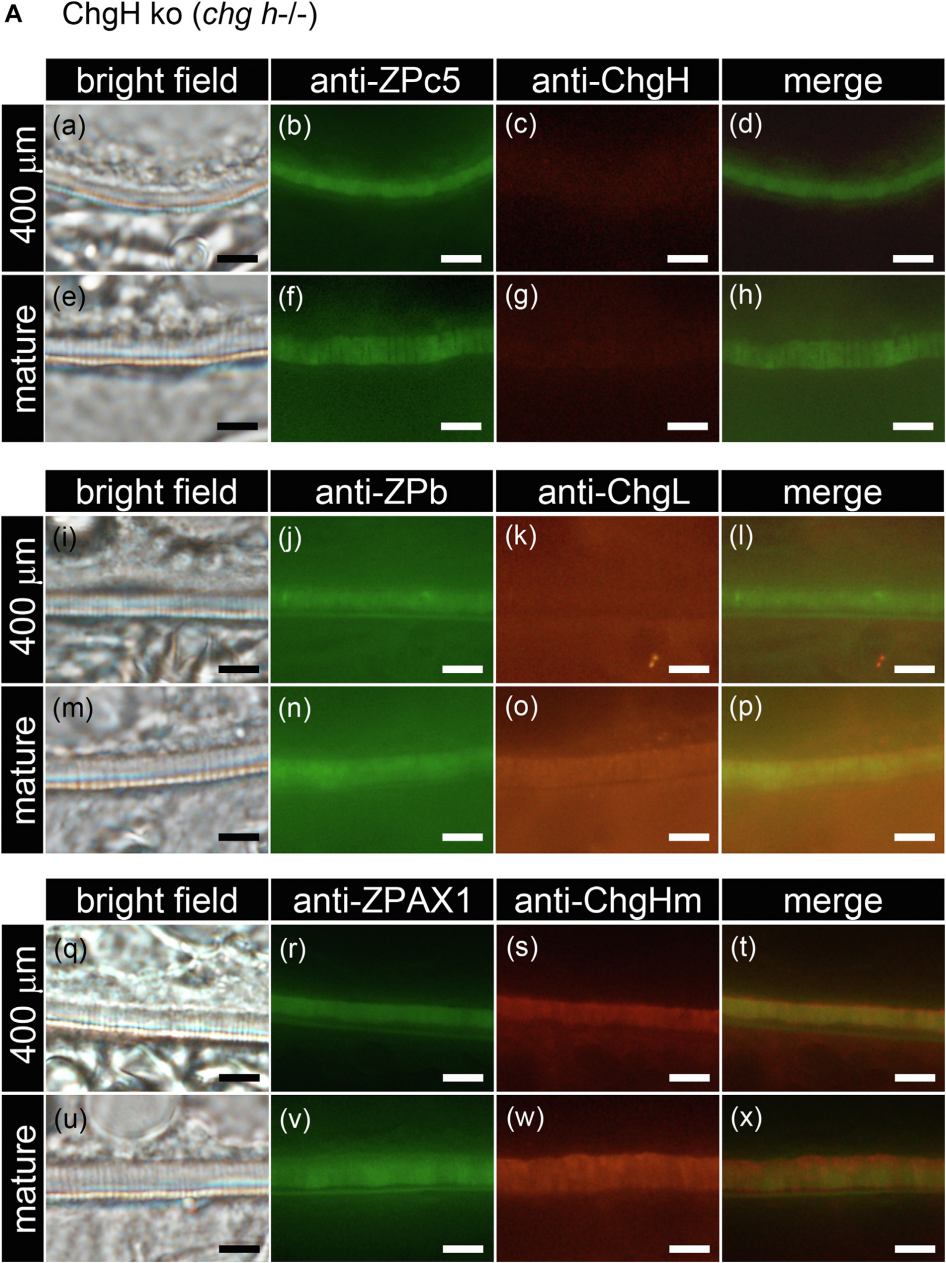

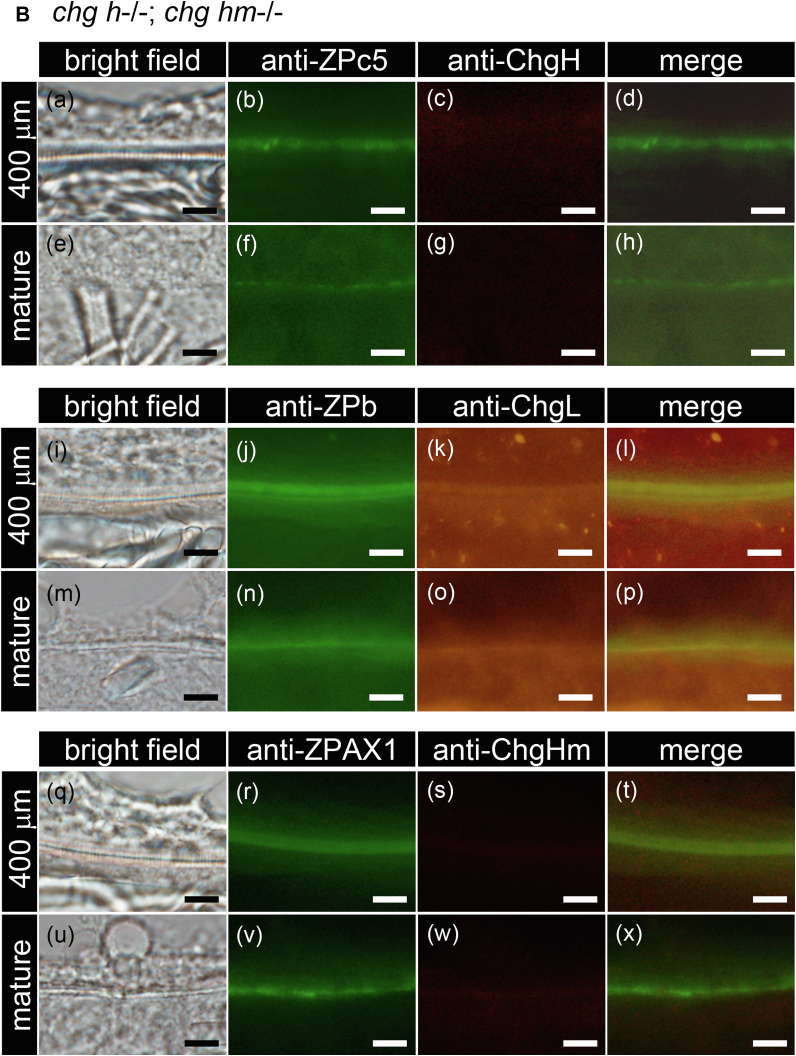

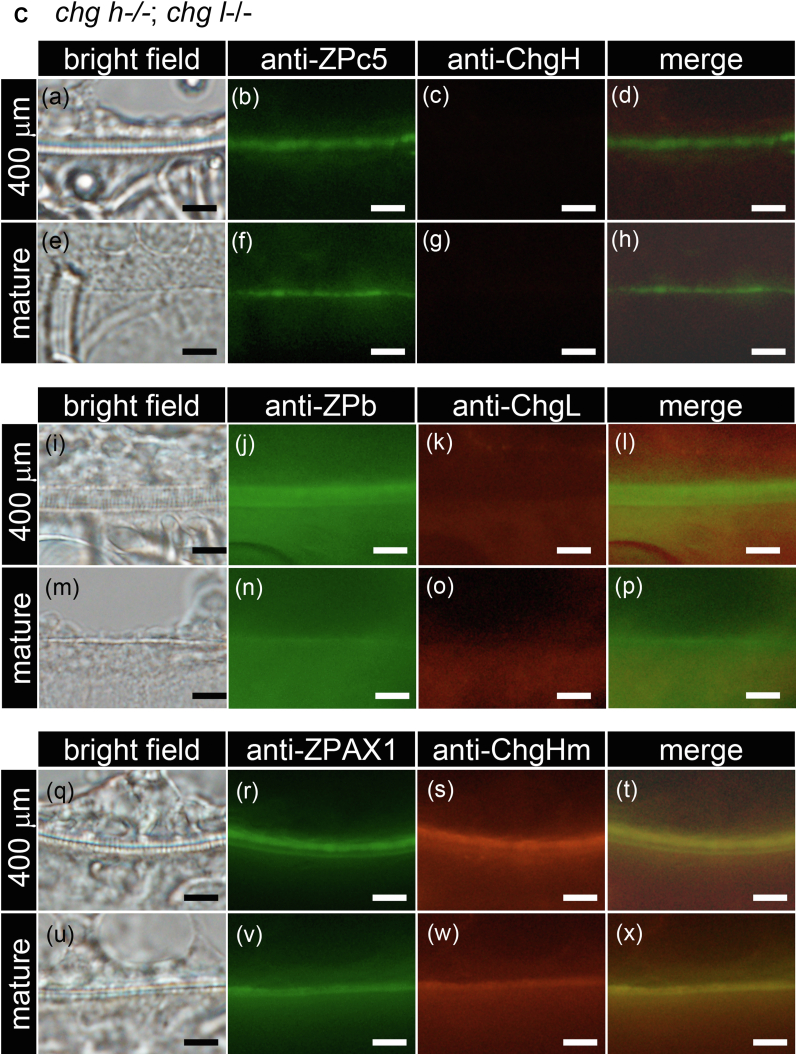
**Localization of each Chg and ZP protein in the egg envelope of the ko growing oocyte.** Localization of each of the Chg and ZP proteins in immature (St. VII, 400 μm diameter) and mature oocytes of ChgH ko (*A*), *chg h*^−/−^; *chg hm*^−/−^ double ko (*B*), *chg h*^−/−^; *chg l*^−/−^ double ko (*C*) was analyzed by double-labeled immunohistochemistry using anti-ChgH and anti-ZPc5 (a–h), anti-ChgL and anti-ZPb (i–p), and anti-ChgHm and anti-ZPAX1 antibodies (q–x). The upper side of all photos is the cytoplasmic side. The scale bars represent 10 μm.

The localization of Chg and the ovary-synthesized ZP proteins in ChgH ko, *chg h*^*−/−*^*; chg hm*^*−/−*^ and *chg h*^*−/−*^*; chg l*^*−/−*^ double ko in egg envelopes was analyzed (Fig. 9, *A*–*C*). The egg envelope of 400-μm-diameter oocytes, corresponding to the stage at which Chg protein accumulation starts in WT medaka, and mature oocytes (Figs. 1 and S1) was analyzed. In ChgH ko, all antibodies except anti-ChgH antibody were detected in the entire range of the egg envelope of oocytes, although the detection of ChgL was very weak (Fig. 9*A*). In mature oocytes of ChgH ko, anti-ChgL and -ChgHm antibodies were detected more on the inner part of the egg envelope, and antibodies against ovary-synthesized ZP proteins were detected more on the outside of the egg envelope. This is noticeable in the merged pictures (Fig. 9*A* (e–h, m–p, u–x)). In *chg h*^*−/−*^*; chg hm*^*−/−*^ and *chg h*^*−/−*^*; chg l*^*−/−*^ double ko, anti-ChgL and -ChgHm antibodies were detected throughout the entire egg envelopes in both stages of oocytes, respectively (Fig. 9*B* (k, o) and Fig. 9*C* (s, w)). In addition, ZPb, ZPc5, and AX1 were localized to the entire egg envelope of immature oocytes, as in WT. However, unlike in WT, they were also localized to the entire egg envelopes in mature eggs (Fig. 9, *A*–*C* (b, f, j, n, r, v)).

## Discussion

### ChgH ko eggs are fertilizable but the polyspermy block system is incomplete

ChgH ko females could not produce normal fertilized eggs by spawning naturally with WT males. The observation suggests that the egg envelope of ChgH ko females was not sufficiently tough, and the egg was collapsed as it passed through the oviduct. In fact, the egg envelope of ChgH ko was only 2.84 ± 0.47 μm thick (Table 1) and the weight-bearing capacity of the egg was 5.7 ± 2.2 g (Fig. 8*B* and Table S2). It was thus easily collapsed by approximately 1/40th of the weight of the WT (219.7 ± 58.0 g). However, when ChgH ko eggs were artificially inseminated with WT sperm, almost all eggs were fertilized. This result suggests that ChgH deficiency does not affect oogenesis and ChgH ko eggs are fertilizable. The fact that the egg envelopes isolated from eggs attached to the abdomen of ChgH ko females by natural spawning were not solubilized by SDS also suggests that the hardening mechanism induced by fertilization worked on ChgH ko egg envelopes. However, many of the fertilized eggs of ChgH ko resulted in abnormal egg cleavage and only a few embryos had normal morphogenesis (Fig. 6, *F* and *H*). All WT eggs artificially inseminated at the same time as ChgH ko eggs resulted in normal morphogenesis (Fig. 6, *E* and *G*). The results suggest that malformation of micropyle, the sperm guidance system employed in teleost fertilization, occurred in thin egg envelope. Interestingly, the egg envelope of zebrafish is approximately 2 μm thick, comparable with that of ChgH ko, but sufficiently tough to endure normal spawning, fertilization, and development. Some fish species have even thinner egg envelopes than this (13). In other words, the fragility of ChgH ko eggs cannot be explained solely by the thinness of their egg envelopes. These results suggest that the egg envelopes of ChgH ko eggs are not thinner simply due to the absence of ChgH but are more fragile due to compositional imbalances.

### ChgH ko and *chg h*^*−/−*^*; chg hm*^*−/−*^ and *chg h*^*−/−*^*; chg l*^*−/−*^ double ko cause extremely thin egg envelopes

ChgHm and ChgL were detected in the egg envelope of ChgH ko by Western blotting and immunohistochemistry, indicating that the egg envelope can form without ChgH. However, the total amount of egg envelope protein per ChgH ko egg was approximately 25% that of the WT, even though ChgH occupies 25 to 30% of the total amount of WT egg envelope protein according to the Coomassie Brilliant Blue–staining SDS-PAGE pattern of UFEs. Since a large amount of Chg proteins was present in serum in spawning medaka (15, 16, 17, 18, 19, 20), a sufficient amount of chgL and chgHm proteins would be supplied to the ovary for choriogenesis in ChgH ko. Therefore, it is unlikely that the thin egg envelope of ChgH ko is caused only by the decrease in Chg protein supplied. Structural analysis of the digestion of the medaka egg envelope by hatching enzymes has revealed that, in the WT egg envelope, the polymerization between ZPB (ChgH and ChgHm) and ZPC (ChgL) occupies the majority, while the polymerization between ZPB and ZPB (ZPC and ZPC) is the minority, suggesting that the binding affinities between ZP domains differ depending on the subtype of ZP proteins (36). This difference in affinity between ZP domains may have thinned the egg envelope of ChgH ko medaka more than the amount of ChgH protein lost. Furthermore, the egg envelopes of *chg h*^*−/−*^*; chg hm*^*−/−*^ and *chg h*^*−/−*^*; chg l*^*−/−*^ double ko were even thinner, approximately 5% and 11% that of WT, respectively. Interestingly, immunohistochemistry results showed that ChgL was polymerized in the egg envelope of *chg h*^−/−^; *chg hm*^−/−^ double ko, in which both major ZPBs were absent. It is possible that ChgL polymerized with the ovary-synthesized ZPb proteins; however, the egg envelope was unable to become thick, since the ZP domains of ChgL inefficiently polymerize with each other.

### The ovary-synthesized ZPs are a “foothold” for egg envelope formation

The thickness of the egg envelopes of ChgH ko and *chg h*^*−/−*^*; chg hm*^*−/−*^ and *chg h*^*−/−*^*; chg l*^*−/−*^ double ko was significantly different from that of WT when compared in mature oocytes but was comparable with WT when compared in immature oocytes (Fig. 7). The egg envelope from double ko medaka was mainly composed of ovary-synthesized ZPb, c5, and AX1 (Fig. 9, *B* and *C*). Immunohistochemical analyses of WT ovaries showed that the egg envelope was initially formed only by ovary-synthesized ZPs (*e.g.*, ZPb, ZPc5, ZPZX1) and it was then quickly thickened by the accumulation of liver-derived Chgs, and finally, most of the egg envelope was occupied by Chgs (Fig. 1). Considering the above results together, the initial egg envelope formation with ovary-synthesized ZP proteins was normal even in ChgH ko or double ko eggs, but the egg envelopes became thinner as the egg diameter increased because they could not thicken sufficiently thereafter due to the Chgs deficiency. Therefore, the initial egg envelope composed of ovary-synthesized ZP proteins works as a foothold for the deposition of liver-derived ZP proteins, since the ZP domains of Chgs are believed to polymerize with those of ovary-synthesized ZP proteins. Interestingly, once Chgs polymerization began, ovary-synthesized ZP proteins were detected in the outermost layers of the egg envelopes of WT medaka. This result suggests that ZP polymerizes in the inward direction of the egg envelope.

### Evolutionary consideration of the egg envelope formation of euteleosts

As mentioned in the introduction, despite the diversity and complexity of ZP gene evolution, only ovary-expressed *zp* genes (*e.g.*, *zpb*, *zpc1-c5*, *zpax1*, *zpax2*) are well conserved in the genomes of all teleosts (14). This study reveals that the ovary-synthesized ZP proteins are the first foothold for egg envelope formation and contribute to egg envelope formation independently of the ZP proteins that are the major components of the egg envelope, such as Chgs. Based on the above results, we inferred the evolutionary history of the egg envelope formation of teleosts. In many vertebrates, the egg envelope is formed by ovary-synthesized ZP proteins. This mechanism is maintained in teleosts. However, the liver-expressed *zp* genes (*chg* genes) emerged from gene duplication and the Chg proteins contributed to the formation of a thick envelope through their deposit in an egg envelope made of ovary-synthesized ZP protein. Interestingly, the N-terminal regions of ChgH and ChgHm are known to be the substrates of transglutaminase responsible for egg envelope hardening and hatching enzymes (30). Therefore, choriogenins, especially ChgH and ChgHm, evolved to contribute to teleost-specific functions. The ovary-expressed *zp* genes are well conserved because they are essential for all teleosts with any type of egg envelope formation mechanism. Actually, in medaka, the results of this study suggested that the ovary-synthesized ZPs is important for the initial process of the egg envelope construction. In this study, we examined the effects of the deficiency of Chg proteins, the major components of the medaka egg envelope, and showed that this resulted in thin and fragile egg envelopes mainly composed of ovary-synthesized ZP proteins. Based on the results of this study, we predict that the loss of ovary-expressed *zp* genes, a minor component but a foothold for egg envelope formation, may render egg envelope formation impossible. This hypothesis will be verified next.

## Experimental procedures

### Animals

All experiments using fish were conducted in accordance with the protocols approved by the Animal Care and Use Committee of Josai University. Wildtype, ChgH ko, *chg h*^*−/−*^*; chg hm*^*−/−*^, and *chg h*^*−/−*^*; chg l*^*−/−*^ double ko d-rR medaka were maintained under a 14-h light and 10-h dark condition at 26 °C.

### Establishment of ko strains by CRISPR/Cas9

ChgH ko, *chg h*^*−/−*^*; chg hm*^*−/−*^, and *chg h*^*−/−*^*; chg l*^*−/−*^ double ko strains were generated by CRISPR/Cas9. The guide RNA (gRNA) of *chg h* (NM_001104807), *chg hm* (NM_001104664), and/or *chg l* (NM_001104803) were designed immediately after the initiation codons (Fig. S2). A mixture of 100 ng/μl gRNA (gRNA), 250 ng/μl Cas9 nuclease, 200 ng/μl tracer RNA, and 0.02% phenol red in phosphate buffered saline (PBS) was injected into the one- or two-cell-stage embryos of d-rR. To generate double ko, two gRNAs were mixed and injected simultaneously. The injected embryos (F0) were crossed with WT d-rR, and the mutations in each target locus were identified by polymerase chain reaction (PCR) (F1). Heterozygous transgene F1 medaka were intercrossed, and homozygous transgenic offspring in which the genotype was checked by sequencing were obtained (F2). Since no ko females spawn spontaneously, the lines after the F3 generation were maintained by mating null mutant males with heterozygous females. F3 and later ko individuals were used in this experiment. The gRNA sequences and primer set for genotyping are listed in Tables S3 and S4.

### Artificial fertilization

Anesthetized mature male WT d-rR were dissected to obtain the testes. Sperm were suspended in 50 μl of fetal bovine serum and placed on ice until use. The ovulated mature eggs were obtained by the dissection of anesthetized WT and each ko strain female d-rR following their placement in 1 ml of 1× BBS (110 mM NaCl, 5 mM KCl, 0.8 mM MgSO_4_, 1.35 mM CaCl_2_, 5 × 10^−5^% phenol red) in a 6-cm dish. A volume of 5 μl of sperm solution was sprinkled on the eggs, and once the formation of the perivitelline space was confirmed, 1× BBS was added in an amount sufficient to soak the eggs. The fertilized eggs were cultured at 26 °C, and beginning the next day, they were cultured in water containing methylene blue.

### Histological analysis of the ovaries

Ovaries dissected from mature females of WT and each ko strain were fixed in 4% paraformaldehyde/PBS and embedded in 5% agarose (Ultra-low Gelling Temperature Agarose, Sigma-Aldrich) supplemented with 20% sucrose. Frozen sections were cut at 12 μm thickness. After removal of agarose, sections were placed in Mayer’s Hematoxylin Solution (FUJIFILM Wako Chemical Co) for 2 min, washed in running water for 15 min, and placed in 0.5% Eosin Y Solution (FUJIFILM Wako Chemical Co) for 2 min. After dehydration with ethanol, they were placed in xylene for 15 min and sealed in LIMO mount (Pharma Co, Ltd).

### Crude purification of medaka hatching enzymes

The hatching liquid of medaka (23) was applied to an S-Sepharose column (GE Healthcare UK Ltd) equilibrated with 50 mM Tris-HCl (pH 8.0) and eluted with 0.5 M NaCl/50 mM Tris-HCl (pH 8.0). The eluted fraction was dialyzed against 50 mM Tris-HCl (pH 8.0) and then stored at 4 °C.

### Measurement of total protein content of egg envelope by Bradford assay

Egg envelopes were isolated from eggs on the abdomen of the natural spawning female of WT, ChgH ko, and *chg h*^*−/−*^*; chg hm*^*−/−*^ double ko. For each egg envelope, 5 μl of crude purified medaka hatching enzyme and 5 μl of 25 mM Tris-HCl (pH 8) were added and incubated at 30 °C for 20 min to completely dissolve the inner layer of the egg envelope. After centrifugation, a dilution series of the supernatant was prepared, Bradford’s reagent (50 mg/l CBB-G250, 5% methanol, 8.5% phosphoric acid) was added, and the absorbance at 595 nm was measured. This experiment was repeated at least three times each in WT and ko egg envelopes (WT: n = 5, ChgH ko: n = 6, *chg h*^−/−^; *chg hm*^−/−^: n = 3,). A calibration curve was prepared with known concentrations of bovine serum albumin.

### Weight-bearing capacity measurement per egg

Eggs on the abdomen of the natural spawning females of WT, ChgH ko, and *chg h*^*−/−*^*; chg hm*^*−/−*^ double ko were collected. One egg was placed in a Petri dish filled with water. The weight was applied from the top of the egg and the weight when the egg was crushed was used as the weight-bearing capacity. This experiment was repeated at least three times (WT: n = 7, ChgH ko: n = 5, *chg h*^−/−^; *chg hm*^−/−^: n = 3).

### Real-time PCR for Chg and ZP gene expression analysis

The females confirmed natural spawning, including collapsed eggs, were designated as mature females. Total RNA was extracted from the ovaries and livers of mature females of WT, ChgH ko, and *chg h*^*−/−*^*; chg hm*^*−/−*^ and *chg h*^*−/−*^*; chg l*^*−/−*^ double ko using the Fast Gene RNA Basic kit (Nippon Genetics Co, Ltd) according to the manufacturer’s instructions. Ovaries contained only St. I-VII oocytes, excluding mature eggs. The extracted total RNA (0.5 μg) served as the template for the synthesis of cDNA using the PrimeScript RT Master Mix (Takara Bio Inc). The *chg h*, *chg hm*, *chg l*, *zpb* (NM_001104747), *zpc1* (NM_001104748), *zpc5* (NM_001104751), and *zpax1* (NM_001104746) transcript levels were determined by quantitative PCR with the LightCycler 96 instrument (Roche Diagnostics). A β-actin gene was used as an internal reference control. The specificity of each primer set (Table S5) was confirmed by a melting curve analysis. Each experiment was performed using RNA extracted from three different individuals (n = 3). The relative expression level of each gene was calculated using the comparative Ct method, with the expression level of each gene in WT set to 1.

### SDS-PAGE and Western blotting of egg envelopes

One-eighth of the WT UFEs and one ChgH ko UFE were loaded onto a 12.5% polyacrylamide gel. The gel was stained by Coomassie Brilliant Blue-250.

One-fortieth of the WT UFEs and half of the ChgH ko UFEs were loaded onto a 12.5% polyacrylamide gel. After SDS-PAGE, proteins were transferred to a polyvinylidene fluoride membrane. The membranes were blocked in TBS containing 1% bovine serum albumin and 0.05% Tween-20 and then incubated with primary antibodies (Table S6). After washing, the membranes were incubated with secondary antibodies (Goat anti-mouse (or -rabbit) IgG alkaline phosphatase (AP) conjugate (Sigma-Aldrich) at 1:2500 dilution, and the alkaline phosphatase activity was visualized by OneStep-NBT/BCIP (Thermo Fisher Scientific Inc).

### Double-label immunohistochemistry of ovarian sections

Frozen sections of 12 μm thickness were prepared as described above. After blocking with PBS containing 2% normal goat serum, the sections were incubated overnight at room temperature with 1:10,000 diluted anti-ChgH, Hm, or L rabbit IgG and 1:250 diluted anti-ZPb, c5, or AX1 mouse IgG in PBS containing 2% normal goat serum (Table S7). For detection of mouse IgG, the sections were incubated with Goat anti-Mouse IgG Biotinylated (Vector Laboratories, Inc) diluted at 1:1000 for 1 h at room temperature, washed with PBS, and incubated with HRP conjugated ABC regent (VECTASTAIN ABC kit, Vector Laboratories, Inc) for another 30 min. Then the washed sections were incubated with Alexa Fluor 488 tyramide (Invitrogen) for 7 min at room temperature. For detection of rabbit IgG, the sections were incubated with Goat anti-Rabbit IgG Alexa Fluor 555 (Invitrogen) diluted 1:1000 for 30 min at room temperature. After washing with PBS, the sections were sealed in CC mount (Diagnostic BioSystems).

### Antibodies

Mouse polyclonal antibodies against ZPb, c5, AX1, and ChgL were generated. The antigen sequences are listed in Table S7. Inclusion bodies of the antigen proteins were harvested from the transformant *Escherichia coli* BL21 Star (DE3) (Thermo Fisher Scientific). Inclusion bodies dissolved in 2M urea/PBS were loaded onto a Ni-NTA Superflow (Qiagen) and purified. Approximately 0.2 mg of each eluted inclusion body solution was injected into Jcl:ICR mice (CLEA Japan, Inc) four times every 2 weeks. Serum samples were harvested from the antigen-injected mice. The serum, diluted to 50% in glycerol with 0.1% sodium azide, was used for immunohistochemistry.

## Data availability

The data that support the findings of this study are available from the corresponding author, K. S., upon reasonable request.

## Ethical statement

The experimental methods were approved by the Ethical Committee of Josai University, and performed in strict conformance with relevant guidelines and regulations.

## Supporting information

This article contains supporting information.

## Conflict of interest

The authors declare that they have no conflicts of interest with the contents of this article.
