# Supplementary material for: Egg envelope formation of medaka Oryzias latipes requires ZP proteins originating from both the liver and ovary
Source: J Biol Chem. 2023 Mar 10;299(4):104600. doi: 10.1016/j.jbc.2023.104600 (PMC10140178; doi:10.1016/j.jbc.2023.104600)
Supplement: Supporting Figures S1 and S2 and Tables S1–S7 [file mmc1.docx]

**Supporting information**

**Egg envelope formation of medaka *Oryzias latipes* requires ZP proteins originating from both the liver and ovary**

**Reo Yokokawa, Kana Watanabe, Shinji Kanda, Yoshihide Nishino, Shigeki Yasumasu, and Kaori Sano**

The supporting information includes additional two figures and seven tables.


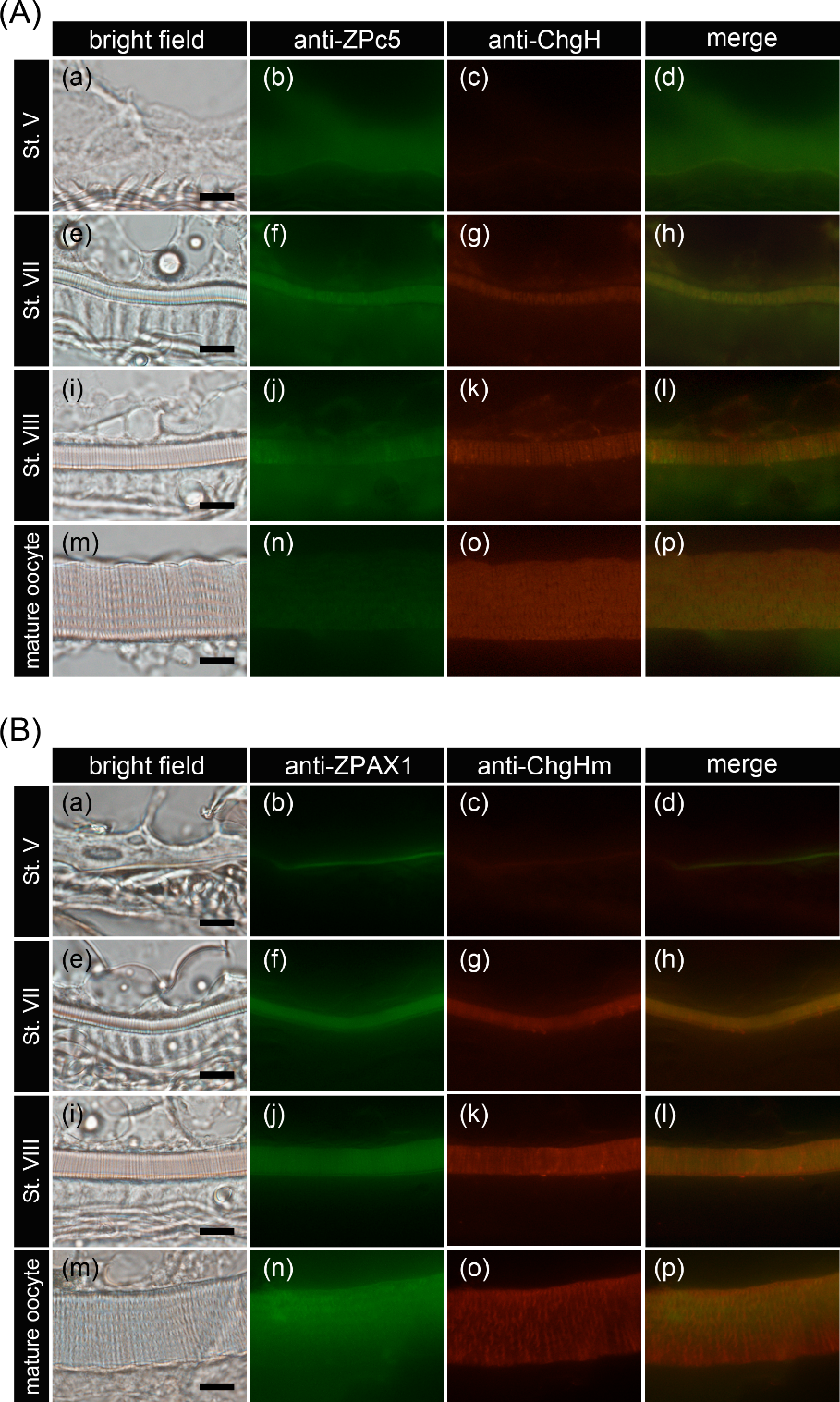


**Figure S1. Localization of ChgH, ChgHm, ZPc5, and ZPAX1 in an egg envelope of the WT growing oocyte.**

The localization of each ZP protein was analyzed by double-labeled immunohistochemistry using anti-ChgH and anti-ZPc5 antibodies (A) and anti-ChgHm and anti-ZPAX1 antibodies (B). (a-d) St. V oocyte (250 μm). (e-h) St. VII oocyte (450 μm). (i-l) St. VIII oocyte (600 μm). (m-p) mature oocyte. The upper side of all photos is the cytoplasmic side. Scale bars: 10 μm.


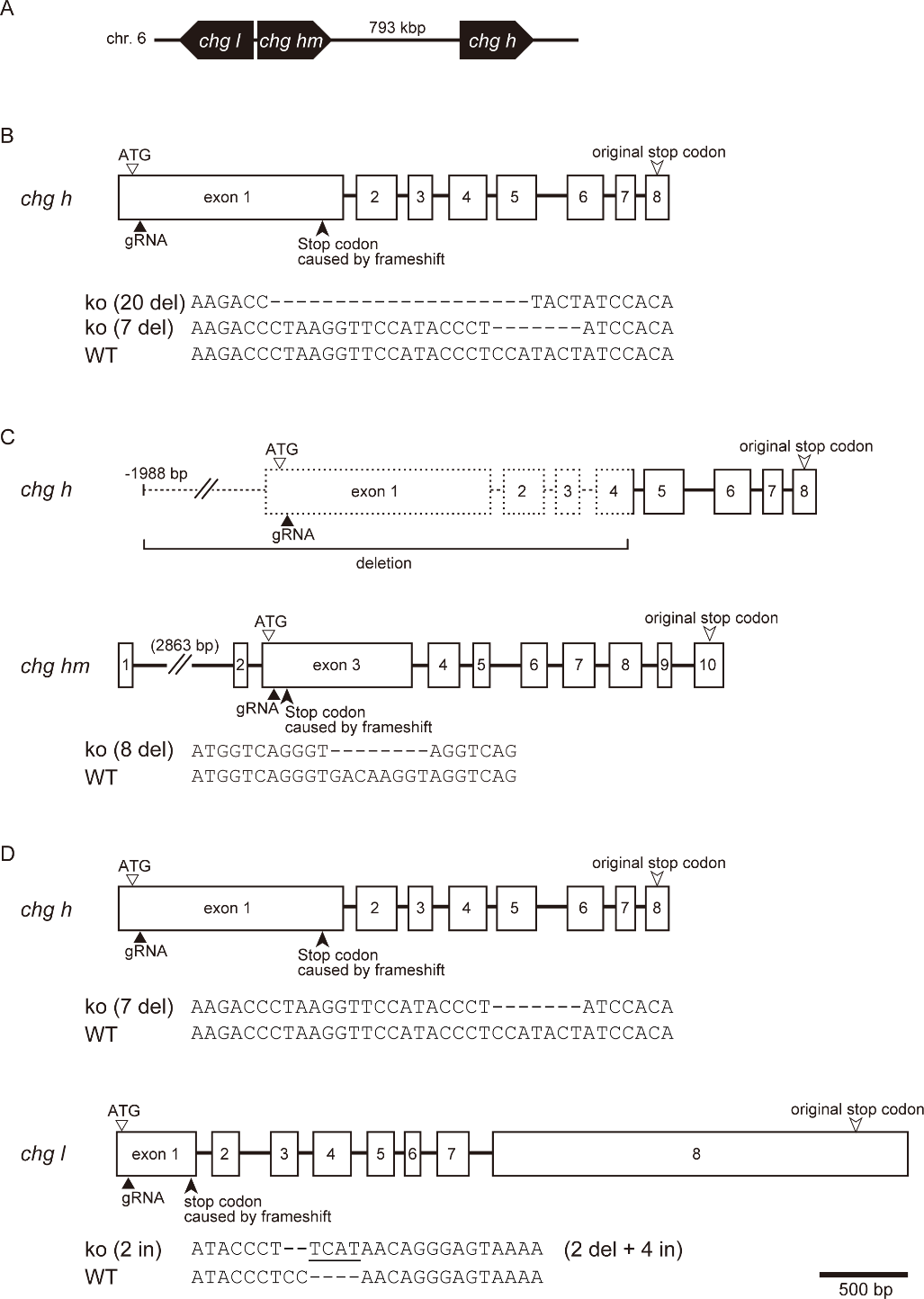


**Figure S2.**

(A) Schematic diagram of the positional relationship between targeted genes in the medaka genome. CRISPR/Cas9 target regions and resulting mutations of (B) ChgH ko (*chg h*^-/-^), (C) *chg h*^-/-^; *chg hm*^-/-^ double ko, and (D) *chg h*^-/-^; *chg l*^-/-^ double ko. Each white box and line indicate an exon and intron, respectively. The downward pointing white triangles and arrowheads indicate the position of the translation start codon and the original stop codon, respectively. The upward pointing black triangles and arrowheads indicate the position of the gRNA and the predicted stop codon caused by the frameshift, respectively. It means the scale bars in E-G.

**Table S1. Artificial insemination of ChgH ko eggs**

**Total eggs** 20

**Fertilizable eggs** 18 (90% of eggs)

Abnormal cell division 17 (94% of fertilized eggs)

embryo axis formation 1 (5.5% of fertilized eggs)

**Table S2. Toughness of egg envelope**

WT (g) ChgH ko (g) *chg h*^-/-^; *chg h*m^-/-^ (g)

#1 274.4 6.5 nd

#2 160.9 4.1 nd

#3 310.2 2.5 nd

#4 194.4 7.8

#5 233.8 7.4

#6 212.7

#7 151.4

**AVG 219.7 5.7 nd**

**SD 58.0 2.2 nd**

**Table S3. gRNA sequences**

gene name gRNA sequence

*chg h* #1 5’-AGGCAACCCTCAAGACCCTAAGG-3’ (20 bp del)

#2 5’-CCCTCCATACTATCCACAGCCGA-3’ (7 bp del)

*chg hm* 5’-GATGGTCAGGGTGACAAGGTAGG-3’

*chg l* 5’-AAACCTTCATACCCTCCAACAGG-3’

**Table S4. Primer sets for genotyping.**

gene name primer sequence

***chg h*** Forward: 5’- TTGAACAGCATCAAAGGTGATGACG -3’

Reverse: 5’- CTCAGAATCCCCAGTATCCTTCGTA -3’

***chg hm*** Forward: 5’- GCAACAAATTGGATGTGAGCAATC -3’

Reverse: 5’- ACATCCTCAACAGCCTCAGCAGC -3’

***chg l*** Forward: 5’- ATGATGAAGTTCACTGCGGTTTGCC -3’

Reverse: 5’- GAGATATCCTGTTCCAGCTGCAACT -3’

**Table S5. Primer sets for real-time PCR.**

gene name primer sequence

***chg h*** Forward: 5’-CCAACTCATCACAGTCGCTTCCT-3’

Reverse: 5’-GTTCACAGCTGACTCCTTGTCCT-3’

***chg hm*** Forward: 5’-CTCCTGGAATCTTGCGTGTGGA-3’

Reverse: 5’-TCAACGTAGACGTTGTCCCTGAGA-3’

***chg l*** Forward: 5’-TGAAGTTCACTGCGGTTTGCCTTGT-3’

Reverse: 5’-CTGGGTGGGATCTTGAGGCGTTT-3’

***zpb*** Forward: 5’-GATGGGTGGAAGTGTCACTATGG-3’

Reverse: 5’-CAGCAGACTGATCGAGTTCACATC-3’

***zpc1*** Forward: 5’-TTGCTTTGGGACATGAGGTGTTC-3’

Reverse: 5’-GCCATTATATGCAGGGTTGTCCA-3’

***zpc5*** Forward: 5’-CTGTGGAAACTCTCCAGTTCAACC-3’

Reverse: 5’-CATGTGATGATCCACCCTGACTGA-3’

***zpax1*** Forward: 5’-TGCCTGAGGATCTCCCTTTTGCC-3’

Reverse: 5’-GTGATGCCCTTGACTGCCGTACT-3’

***β-actin*** Forward: 5’-ATCCTGCGGTATCCATGAGACCA-3’

Reverse: 5’-GGGCTGTGATCTCCTTCTGCATTC-3’

**Table S6. Antibodies for western blotting.**

name host species antigen sequence

anti-ChgH Rabbit 327-629 (AAM47576)

anti-ChgHm Rabbit 556-572 (NP_001098134)

anti-ChgL Mouse 22-141 (NP_001098273)

**Table S7. Antibodies for double labeled immunohistochemistry**

name host species antigen sequence

anti-ChgH Rabbit 327-629 (AAM47576)

anti-ChgHm Rabbit 26- 39 (NP_001098134)

anti-ChgL Rabbit 142-390 (NP_001098273)

anti-ZPb Mouse 21-166 (NM_001104747)

anti-ZPc5 Mouse 62-180 (AF331675)

anti-ZPAX1 Mouse 280-514 (NM_001104746)
